# An expeditious and environmentally benign synthesis of dispiro-3-phenylpyrrolothiazoles in ACI/EG eutectic mixture and its antioxidant and antimicrobial activities against urinary tract pathogens

**DOI:** 10.1186/s13065-019-0553-3

**Published:** 2019-03-28

**Authors:** Govindasami Periyasami, Karuppiah Ponmurugan, Natarajan Arumugam, Raju Sureshkumar, Mostafizur Rahaman, Durairaju Periyan, Naif Abdullah Al-Dhabi, Shaykha Alzahly, Ali Aldalbahi

**Affiliations:** 1 Department of Chemistry, College of Science, King Saud University, P.O. Box 2455, Riyadh, 11451 Saudi Arabia; 2 Department of Organic Chemistry, University of Madras, Guindy Campus, Chennai, 600 025 India; 3 Department of Botany and Microbiology, College of Science, King Saud University, P.O. Box. 2455, Riyadh, 11451 Saudi Arabia; 4 Department of Chemistry, Thiruvalluar Government Arts College, Periyar University, Rasipuram, India

**Keywords:** Eutectic mixture, ACI/EG, Green protocol, Dispiro-3-phenylpyrrolothiazole, Uropathogens

## Abstract

The present study reveals the robust and facile methodology for the synthesis of massively selective dispiro-3-phenylpyrrolothiazole hybrids via one-pot 1,3-dipolar cycloaddition reaction by environmentally supported solvents and to evaluate their biological activities. The quaternary ammonium salt eutectic mixture, acetylcholine iodide-ethylene glycol (ACI/EG) medium brings an efficient environment for the synthesis of dispiropyrrolothiazole with excellent yield in shorter reaction time than imidazolium ionic liquids. The eutectic mixture was recovered and reused without any significant drop in their catalytic activity. Among the eight synthesized compounds **4a–h**, halogen derivatives are exhibiting significant antimicrobial activities against selected uropathogens pathogens. Interestingly, chloro and bromo derivatives exhibits the minimum inhibitory concentration (MIC) of 12.5 μg/ml and 6.25 μg/ml towards *Escherichia coli, Klebsiella pneumonia,* and *Staphylococcus aureus* respectively. In addition, the IC_50_ values of DPPH radicals with synthesized compounds are interesting, particularly compounds **4a**, **4d** and **4e** shows lower than the control BHA indicating their potent scavenging ability of free radicals.

**Electronic supplementary material:**

The online version of this article (10.1186/s13065-019-0553-3) contains supplementary material, which is available to authorized users.

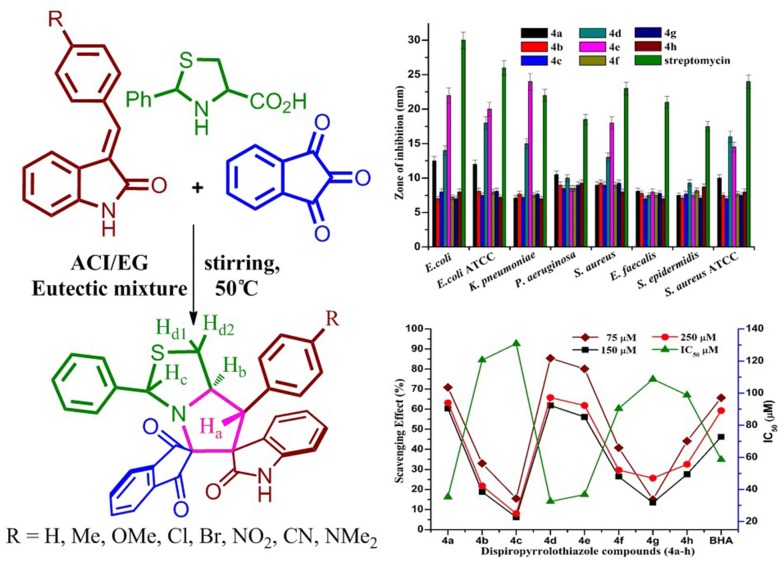

## Introduction

Adult women become frequent victims than men at a ratio of 8:1 for urinary tract infection (UTI) because of their anatomical differences. In general, Gram-positive and Gram-negative bacteria and certain fungi are the causative agents of UTI. Frequent infection is the most important factor contributing to high risk UTI which results in pyelonephritis with sepsis, renal damage, kidney damage, bladder inflammation and urethritis. By nature, urinary track is sterile but colonization of pathogens, particularly Uropathogenic *Escherichia coli* (UPEC) in the urinary tract will leads to highly complicated UTI [1]. The pathogens viz. *Staphylococcus saprophyticus*, *Enterococcus faecalis*, *Pseudomonas aeruginosa* and *Candida* spp. may create non-complicating infections in the initial stage. Unfortunately, followed by UPEC infection, *Enterococcus* spp., *Klebsiella pneumoniae*, *Candida* spp., *Staphylococcus aureus*, *P. aeruginosa*, *P. mirabilis*, and group B *Streptococcus* (GBS) are the responsible pathogens for complicated UTI. UTI is more precarious at the time of pregnancy for both parental and infant health because it can affect kidney easily [2]. Nosocomial infections [3, 4] and increasing resistance to antimicrobial agents [5] are the other unavoidable risk factors associated with UTI treatment. Though antibiotics can be used to treat UTI, long-term treatment creates antibiotic resistance which leads to negative side-effect of UTI treatment including exhaustion of beneficial gut and mucosal microorganism, hypersensitivity, and suppressing immune development in the body [6]. Certainly, there is a need to develop a highly potent and efficient antimicrobial drug for the treatment of infectious diseases which is suitable to treat patients.

Heteroatom consisting cyclic hydrocarbons are fascinating molecules because of their treasured applications in medicinal field. Particularly, structurally rigid spiro heterocyclic analogues showed highly pronounced pharmacological properties and exist in many naturally occurring alkaloids [7]. Having medicinal values, thiazolidine ring systems play an important role in organic synthesis particularly antimicrobial substances such as penicillins, cephalosporins, narcodicins, thienamicyn and other compounds that have physiological activities have been prepared from thiazolidine [8]. It is noted that spiropyrrolothiazole analogues are interesting because of their wide range of biological activities such as anti-cancer [9], anti-diabetic [10], antibiotic [11], anti-inflammatory [12], hepatoprotective [13], anti-convulsant [14], anti-leukemic agents [15], Alzheimer disease [16], and also good in anti-mycobacterial [17]. Spiro indolin-2-one nucleus was reported to treat diabetic patients, HIV-1 protease inhibitors, potent gastrain/CCK-B receptor antagonist and for growth hormone secretagogue receptor agonists [18]. Also, indane-1,3-dione displays anti-blood coagulation, anti-inflammatory, anti-biotic and anti-convulsant activities [19]. Synthesis and antimicrobial screening of a series of structurally complexed molecules with the above mentioned molecular units viz. indane-1,3-dione, indolin-2-one, and pyrrolothiazole ring with spiro junction will be a novel compound with efficient antimicrobial activities against UTI.

The design of present molecular framework stems from the promising UTI activity of thiazolidine units reported by Reese et al. [20]. In addition, the supposition that the rigidity of spiro molecular frameworks would easily binds with the biomolecules, prompted us to synthesize spiropyrrolothiazolidine derivatives for UTI applications. In these contexts, multicomponent 1,3-dipolar cycloaddition reactions are highly suitable and powerful methodology for the construction of pharmacologically valuable heterocyclic compounds. Particularly the chemistry of azomethine ylide 1,3-dipolar cycloaddition reaction is an interesting, less time consuming, and significant for construction of heterocyclic compounds with high stereo and chemoselectivity [21, 22]. Considering framework of environmental green chemical approach, alternative medium for organic solvents occupying predominating place in the organic synthesis. The required reaction medium must have environmentally benign criteria such as non-toxicity, biodegradability, availability, recyclability and also in economically beneficial among others. However, interesting green protocols have been reported in the literature for the synthesis of heterocyclic compounds through multicomponent reaction including microwave assisted [23], solid support and montmorillonite clay catalyzed [24, 25], ultrasonic triggered [26], and with different class of eutectic mixture solvent mediated synthesis [27, 28]. Among the various green protocols, room temperature eutectic mixtures are perspective and effective method for chemical transformations.

Environmentally green quaternary ammonium salt eutectic mixture, Acetylcholine iodide-ethylene glycol (ACI/EG) mediated protocol is an efficient reaction medium for the synthesis of heterocyclic molecules through multicomponent reaction methodology has been reported by our research group recently [29]. In continuous of our research program on the synthesis of spiro heterocyclic derivatives, for the first time, herein we wish to report that ACI/EG as an efficient eutectic mixture medium for the synthesis of biologically significant dispiro-3-phenylpyrrolothiazole analogues and their UTI activities.

## Results and discussion

### Chemistry

Cyclization of l-cysteine with benzaldehyde in water medium undergoes a smooth reaction yielding an analogue of proteinogenic amino acid, 2-phenyl-1,3-thiazolidine-4-carboxylic acid (**1**) at room temperature [30] which is used to generate in situ azomethine ylide with ninhydrin (**2**). The dipolarophile bearing indolin-2-one group was prepared by reacting oxindole with various *p*-substituted aromatic aldehyde through base catalyst condensation reaction. In the present investigation in a green protocol, the cyclic amino acid, 2-phenyl-1,3-thiazolidine-4-carboxylic acid (**1**) was reacted with triketone, ninhydrin (**2**) to generate in situ azomethine ylide, which undergoes one-pot 1,3-dipolar cycloaddition with various *para*-alkyl/halide substituted 3-arylideneoxindoles **(3a–h)**, as dipolarophiles under optimized reaction conditions. In the initial stage, a pilot reaction was carried out with cyclic amino acid (**1**), ninhydrin (**2**), and 3-benzylideneindolin-2-one **(3a)** by using imidazolium eutectic mixtures viz., [Bmim][OH], [Bmim][Br], [Bmim][BF_4_], [Bmim][PF_6_], [Bmim][Cl], [Emim][ClO_4_], [Emim][CF_3_SO_3_], [Emim][PF_6_], [Emim][NO_3_] and also in quaternary ammonium salt eutectic mixture ACI/EG as solvent. Among the various solvent medium, our recent research [29, 31] heads-up us to utilize ACI/EG eutectic mixture as a green solvent reaction medium and it furnished dispiro-3-phenylpyrrolothiazole **4a** in excellent yield of 89% in short reaction time at 50 °C. As eutectic mixture possesses similar physio-chemical properties as ionic liquids [32], the rationale for the formation of spirocycloadduct follows the same pathway as in ionic liquids [33, 34]. Optimization of the reactions in room temperature and 50 °C were compared and presented in Table 1. The required inexpensive quaternary ammonium salt eutectic mixture, ACI/EG was prepared in good yield by mixing acetylcholine iodide and ethylene glycol at a 1: 9 molar ratio and then the mixture was heated at 70 °C [35].

**Table 1:** Optimization of the synthesis of dispiropyrolothiazole **4a**

Entry	Solvent	Time (h)	Yield (%)	
			RT	50 °C
1	[Bmim][OH]	4	25	45
2	[Bmim][Br]	4	30	48
3	[Bmim][Cl]	4	30	46
4	[Bmim][BF_4_]	4	32	46
5	[Bmim][PF_6_]	4	28	49
6	[Emim][ClO_4_]	4	32	46
7	[Emim][CF_3_SO_3_]	4	46	52
8	[Emim][PF_6_]	4	38	58
9	[Emim][NO_3_]	4	30	42
10	ACI/EG	1	78	89

**Scheme 1:**
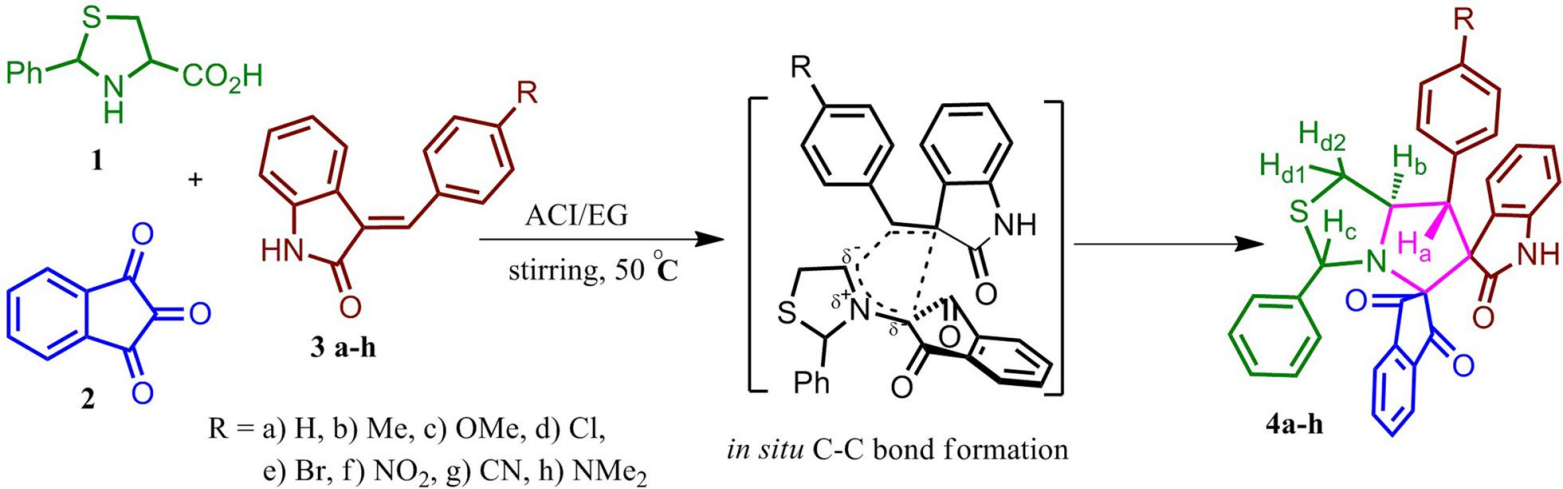
Synthesis of indanedione and oxindole tethered dispiropyrrolothiazole hybrids

Having optimized reaction conditions, all the subsequent reactions were performed through decarboxylative azomethine ylide condensation of an equimolar mixture of the reactants **1**, **2** and **3a–h** in one-pot treated in ACI/EG eutectic mixture at 50 °C for 1 h (Scheme 1). After completion of the reaction (monitored by TLC), the crude product was washed with water and the pure novel dispiro-3-phenylpyrrolothiazoles (**4a–h**) was isolated through flash column chromatography by using EtOAc:hexane (2:8).

**Fig. 1:**
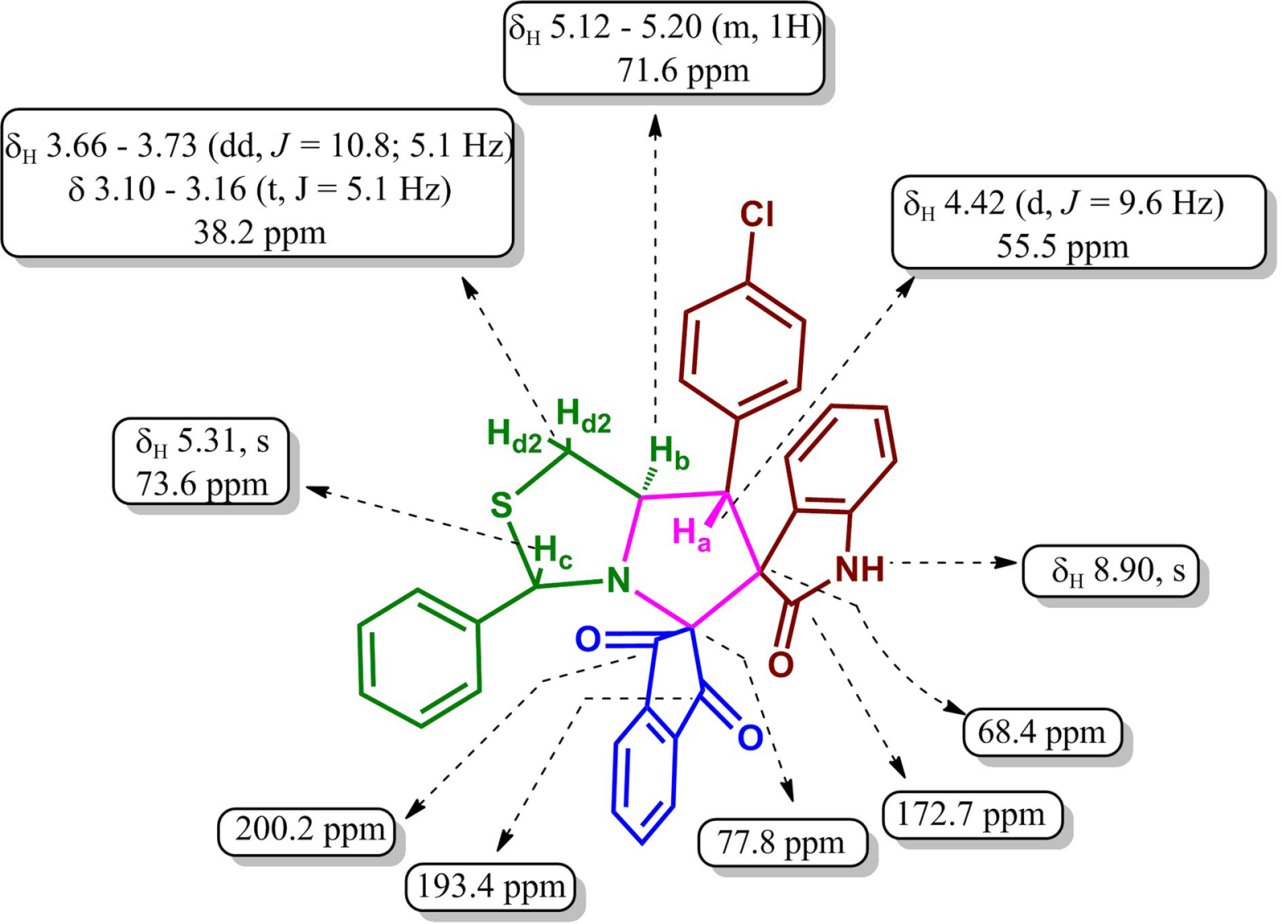
Selected chemical shift values of dispiropyrrolothiazole **4d**

The structure and stereochemistry of all the cycloadducts were confirmed by their spectral data. In the **IR** spectrum of cycloadduct **4d**, the carbonyl groups of indane-1,3-dione exhibited two absorption bands at 1740 cm^−1^ and 1703 cm^−1^ and the amide carbonyl carbon exhibited a band at 1695 cm^−1^. ^1^H NMR spectrum of compound **4d** showed a doublet at δ 4.42 for H_a_ proton and a multiplet at δ 5.12–5.20 for H_b_ proton and their correlation was also evidenced by ^1^H-^1^H COSY spectrum. The benzylic proton of the thiazolidine ring resonated as a singlet at δ 5.31 and the corresponding carbon signal appeared at 73.6 ppm as evidenced from its ^1^H-^13^C COSY by the appearance of isolated contour plot. The two multiplets appeared at δ 3.10–3.16 and δ 3.66–3.73 were due to H_d_ protons. In hetero-COSY spectrum, the carbon corresponding to these H_d_ protons appeared at 38.2 ppm, which is further confirmed from DEPT-135 analysis. The H_b_ proton in tertiary carbon appeared as a multiplet between δ 5.12 and 5.20. The NH proton of the oxindole ring system resonated as a singlet at δ 8.90. In the ^13^C NMR spectrum of cycloadduct **4d**, two spiro quaternary carbons appeared at 68.40 and 77.85 ppm. The oxindole carbonyl carbon resonated at 172.76 ppm and indanedione carbonyl carbons appeared at 193.44 and 200.20 ppm. For further clarification, 1D and 2D NMR spectra of compound **4d** is provided in the Additional file 1. Finally, the mass spectrum of cycloadduct **4d** exhibited the molecular ion peak at *m/z* 563.07 which confirmed the formation of cycloadduct and the compound gave satisfactory elemental analysis. All the spectral data agreed well with the deduced structure of the cycloadduct. The data in Table 2 show that the ability of formation of cycloadducts in the presence of either electron releasing or electron withdrawing groups at the dipolarophile. The pictorial representation of the chemical shift values for cycloadduct **4d** is presented in Fig. 1.

**Table 2:** Yields and melting points of dispiropyrrolothiazole hybrids **4a–h**

Entry	Compound	Mp (°C)	Yield (%)^a^
1.	**4a**	132–134	89
2.	**4b**	122–124	90
3.	**4c**	128–130	92
4.	**4d**	140–142	88
5.	**4e**	138–140	86
6.	**4f**	152–154	85
7.	**4g**	112–114	85
8.	**4h**	166–168	90

^a^Reactions were carried out 50 °C and the isolated yield of the product was purified by flash column chromatography

**Fig. 2:**
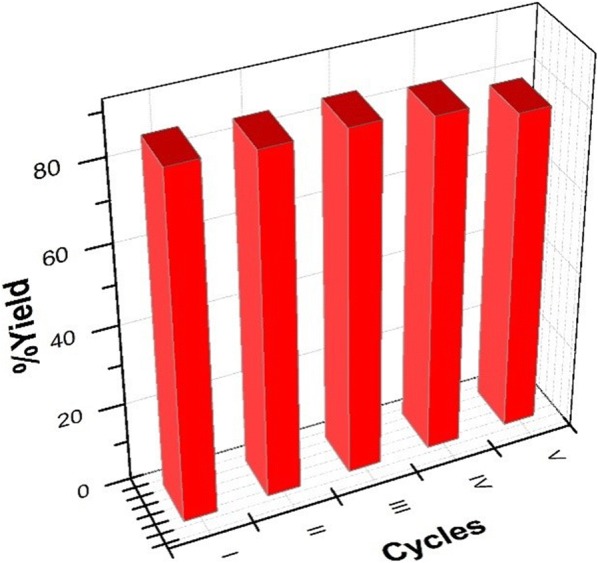
Efficiency of the recovered eutectic mixture ACI/EG at 50 °C for the synthesis of **4a**

The recovered wet eutectic mixture ACI/EG from the reaction mixture was completely purified by applying high vacuum at 60 °C. The catalytic activity of the purified eutectic mixture was further investigated and identified it efficiency for consecutive reaction. The results presented in Fig. 2, clearly show that the catalytic activity and efficiency of the reused eutectic mixture are good and effective for four consecutive runs.

### Antimicrobial activity

**Fig. 3:**
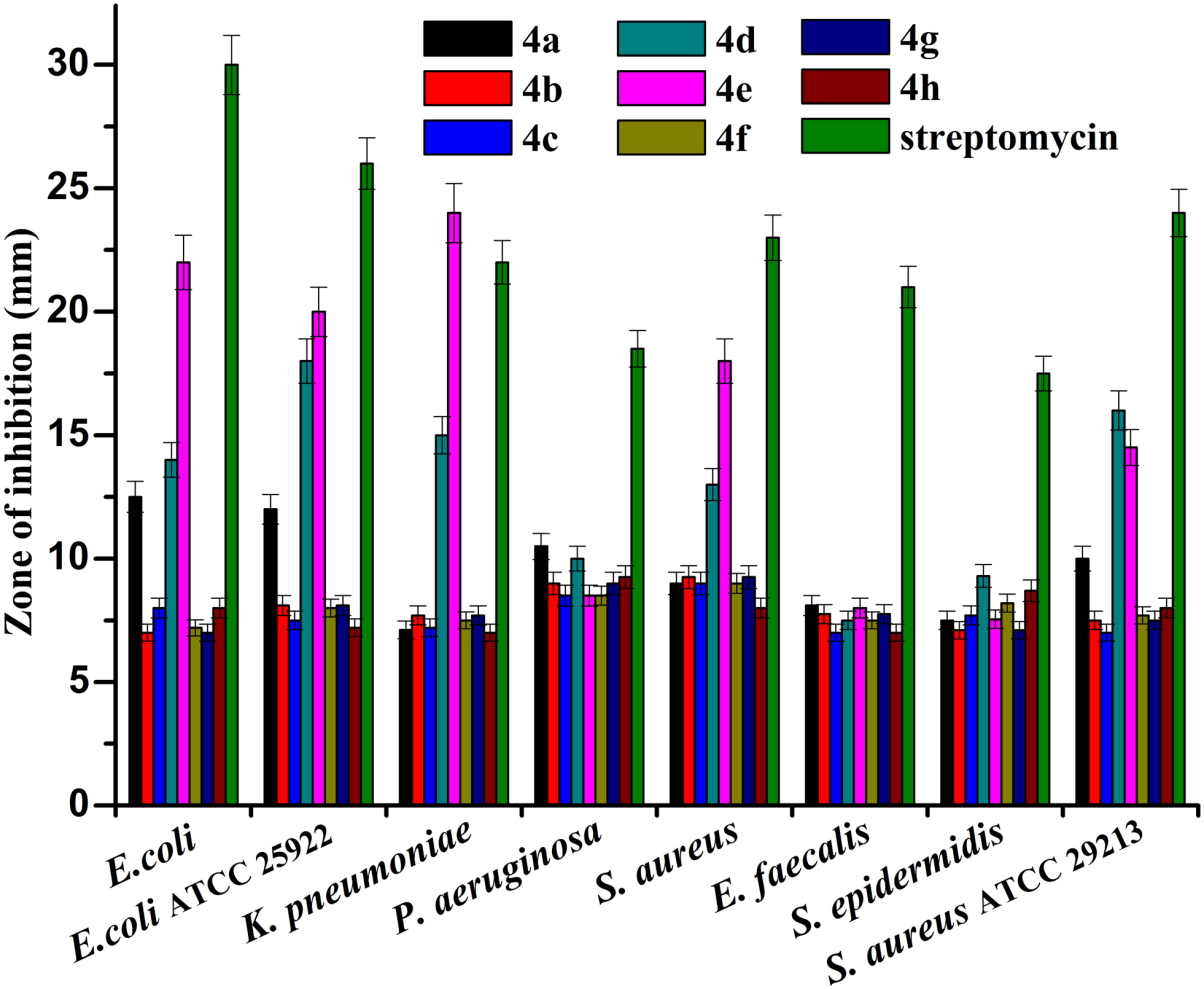
Antibacterial activity of dispiropyrrololhiazoles **4a–h** against uropathogens

In the biological studies, the antibacterial activity assessment of dispiropyrrolothiazole compounds **4a–h** against the three Gram-negative and three Gram-positive uropathogens and two ATCC reference strains were studied using the agar well diffusion method. The data accompanying with the antibacterial prospective of the dispiropyrrolothiazole compounds are presented in Table S1 in the Additional file 1. The efficacy of antibacterial inhibitory activity diverges with respect to each compound against a panel of uropathogens. The zone of inhibition diameters was a maximum of 26.00 mm and a minimum of 9.00 mm whereas the standard antibiotic streptomycin had a higher zone of inhibition ranging from 15.00 to 30.00 mm (Fig. 3). The dispiropyrrolothiazole compounds exhibited antibacterial activity against almost all tested uropathogens except *E. faecalis*. The highest inhibition zone was observed on **4e** against *K. pneumoniae* (24.00 mm) and *E. coli* (22.00 mm) followed by *S. aureus* (18.00 mm). The moderate inhibitory activity was displayed by **4d** *K. pneumoniae* (15.00 mm) and *E. coli* (14.00 mm) followed by *S. aureus* (13.00 mm) and *P. aeruginosa* (10.00 mm) and **4a** *E. coli* (12.50 mm), *S. aureus* (9.00 mm) and *P. aeruginosa* (10.5 mm)) was found to be less effective than compound **4d**. On the other hand, other dispiro-3-phenylpyrrolothiazole derivatives such as **4b**, **4c**, **4f**, **4g** and **4h** showed least efficacy (7.10 to 9.50 mm) against tested uropathogens. Among all tested synthetic dispiro-3-phenylpyrrolothiazole analogues, **4a**, **4d** and **4e** were more efficient and the order of potential antibacterial activity is **4e **> **4d **> **4a**.

The MIC test of dispiropyrrolothiazole derivatives against six uropathogens and two ATCC strains were carried out with the tube dilution technique. The MIC results are shown in Table 3. The half of tested uropathogens was showed resistant to compound **4a**, later it was exempted from MIC study. The MIC values were range from 6.25 to 100.00 µg/ml. The most susceptible uropathogens were *K. pneumoniae* and *E. coli* (6.25 µg/ml), followed by *S. aureus* (12.50 µg/ml), whereas the other uropathogens MICs range from 25.00 to 100.00 µg/ml and it was deliberately less susceptible (Table 3).

**Table 3:** MIC of dispiropyrrolothiazole derivatives **4d** and **4e** against uropathogens

UTI bacterial pathogens	MIC range (µg)	
	**4d**	**4e**
*E*. *coli*	25.00	6.25
*E*. *coli* ATCC 25922	12.50	12.50
*K. pneumoniae*	12.50	6.25
*P*. *aeruginosa*	50.00	100.00
*S*. *aureus*	25.00	12.25
*S. epidermidis*	100.00	100.0
*S*. *aureus* ATCC 29213	12.50	6.25

### Antioxidant activity by DPPH method

**Fig. 4:**
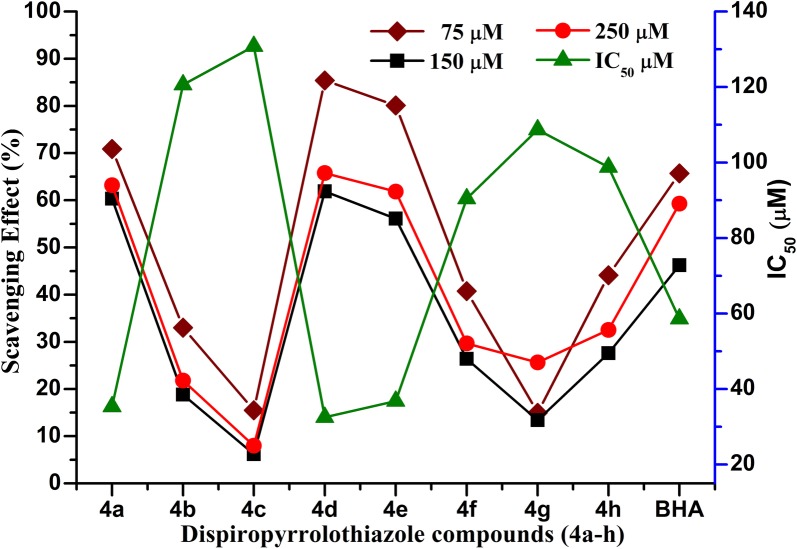
Antioxidant activity of dispiropyrrololhiazole compounds **4a–h** by DPPH method

The free radical scavenging activity of dispiropyrrolothiazole compounds **4a–h** was carried out in the presence of 1,1-diphenyl-2-picrylhydrazyl (DPPH- free radical) and using *tert*-butyl-4-hydroxyanisole (BHA) antioxidant agents as positive control. The DPPH method is highly reliable, rapid and also one of the most appropriate methods for determines the antioxidant activity. The inhibitory effects of different concentrations of synthesized dispiropyrrolothiazole compounds **4a–h** on DPPH radical are depicted in Fig. 4. The antioxidant activity is expressed in terms of % inhibition and IC_50_ (effective concentration for scavenging 50% of the initial DPPH) value (μM).

Based on the results, among all the synthesized dispiropyrrololhiazoles compounds **4a–h** showed scavenging activity towards DPPH. These compounds were shown an active inhibitory effect against DPPH radical at 250 μM concentration and inhibition rates were: 97.20% ± 1.05% (for **4d**), 94.00% ± 1.25% (for **4a**), and 92.30% ± 1.50% (for **4e**) better than the positive control BHA (89.10% ± 1.30%). Whereas, the compounds **4b, 4c** and **4** **g** were exposed less inhibitory activity than the BHA. These compounds **4a, d, e** inhibited the DPPH activity with an IC_50_ = 35.30 μM (**4a**), 32.50 μM (**4d**) and 36.80 μM (**4e**) which is better than the specific inhibitor of BHA (IC_50_ = 58.60 μM).

## Materials and methods

### Bacterial strain

The uropathogens being used were *Escherichia coli* (*E. coli*), *Pseudomonas aeruginosa* (*P. aeruginosa*), *Klebsiella pneumoniae* (*K. pneumoniae*), *Staphylococcus aureus* (*S. aureus*), *Staphylococcus epidermidis* (*S. epidermidis*) and *Enterococcus faecalis* (*E. faecalis*). In addition, *Escherichia coli* (*E. coli* ATCC 25922) and *Staphylococcus aureus* (*S. aureus* ATCC 29213), the two ATCC reference cultures, are also included. The bacterial cultures were obtained from BioLine Laboratory, Coimbatore, Tamil Nadu. The organisms were periodically sub-cultured and maintained in a nutrient agar slant at 4 °C.

### Inoculum preparation

The uropathogens were grown in a 5 ml brain heart infusion (BHI) broth at 37 °C antibacterial activity assessment. Eighteen hour old pure bacterial culture was used to prepare a density of 10^8^ cells/ml of 0.5 McFarland standard at the time of each experiment. Muller-Hinton agar (MHA) was prepared according to the manufacturer’s instruction, autoclaved and dispensed in a sterile plate. All the culture media were purchased from HiMedia Pvt. Ltd., Mumbai, India.

### Antibacterial susceptibility tests

#### Agar well diffusion

The UTI bacterial broth culture was prepared to a density of 10^8^ cells/ml of 0.5 McFarland standards. The aliquot was spread evenly onto Muller Hinton Agar plates with a sterile cotton swab. Then, the plated medium was allowed to dry at room temperature for 30 min [36]. On each plate, equidistant wells were made with a 6 mm diameter sterilized, cork borer, 2 mm from the edge of the plate. Fifty microliter of each dispiro-3-phenylpyrrolothiazole compounds (100 µg/ml) were aseptically introduced into an agar well. Streptomycin (15 µg/ml) were used as positive controls and the DMSO was included as negative controls. This was followed by allowing the agar plate on the bench for 20 min pre-diffusion followed by incubation at 37 °C for 24 h. The formation of a clear inhibition zone of ≥ 7 mm diameters around the wells was regarded as significant susceptibility of the organisms to the dispiro-3-phenylpyrrolothiazoles **4a–h**. All the experiment was performed in triplicate.

#### Determination of minimum inhibitory concentration (MIC)

The tube dilution technique [37] was used to determine the MIC of dispiro-3-phenylpyrrolothiazole derivatives that shows a maximum zone of inhibition at agar well methods. The dispiro-3-phenylpyrrolothiazole derivatives **4d** and **4e** was used to determine the MIC by tube dilution technique which was showed maximum zone of inhibition at agar well method. Both compounds were serially diluted in the range from 3.125 to 100 µg/ml. The tubes were inoculated with 100 µl of UTI bacterial pathogens at a concentration of 10^6^ cells/ml. Standard antibiotics streptomycin was included in the assay for comparison. Nutrient broth with the inoculum only was used as control. All the experiments were carried out in triplicate. The tubes were incubated aerobically at 37 °C for 18 h. The growths of inoculum were decreased in the next dilution was taken as MIC values.

#### Antioxidant activity by DPPH method

The antioxidant activity of all the synthesized compounds was evaluated by DPPH method with some modifications and compared with standard BHA. The 400 μM solution of DPPH (2 ml) in ethanol was added to tested sample solutions (2 ml) of different concentrations (75, 150, and 250 μM) in acetone–ethanol 4:96 *v*/*v*. The samples were kept in the dark at room temperature. After 30 min the absorbance values were measured at 517 nm and were converted into the percentage antioxidant activity (%) using the formula [38]:

1 $$\% \, = \, \{ 1 { } - \, \left[ {\left( {{\text{A}}_{\text{sample}} - {\text{ A}}_{\text{sampleblank}} } \right)/{\text{A}}_{\text{control}} } \right] \, \times { 1}00$$

where, A_control_ was the absorbance of DPPH solution without sample, A_sample_ was the absorbance of sample solution with DPPH, A_sampleblank_ was the absorbance of the sample solutions without the DPPH.

All analyses were undertaken on three replicates and the results averaged. The IC_50_ values were calculated by linear regression plots, where the abscissa represented the concentration of tested compound solution (75, 150, and 250 μM) and the ordinate represented the average percent of antioxidant activity from three separate tests. The absorbance was measured on a spectrophotometer.

## Experimental

### General procedure for the synthesis of dispiropyrrolothiazoles **4a–h**

To a suspension of 2-phenylthiazolidine-4-carboxylic acid (1) (209 mg, 1.0 mmol) in ACI/EG (3.0 ml) were added ninhydrin (2) (160 mg, 1.0 mmol) and 3-arylidene isatin (3a–h) (1.0 mmol) at room temperature. The reaction mixture was stirred at 50 °C for 1 h. After completion of the reaction, tested by TLC, the crude product was washed with water and purified by flash column chromatography over silica gel with a hexane–ethyl acetate mixture (8:2) to give pure dispiropyrroloisoquinolines (4a–h) in good to excellent yield.

### Spectral data of the synthesized cycloadducts

Hexahydro-3,7-diphenylspiro[5.2′]-2*H*-indene-1′,3′-dione-spiro[6.3″] oxindolopyrrolo[1,2-*c*]thiazole, 4a.Yellow solid, mp 132–134 °C; IR (KBr) 3388, 1744, 1706, 1698 cm^−1^; ^1^H (300 MHz, CDCl_3_) *δ* 3.13–3.17 (m, 1H, H_d1_); 3.65–3.71 (m, 1H, H_d2_); 4.37 (d, 1H, H_a_, *J* = 9.3 Hz); 5.16–5.23 (m, 1H, H_b_); 5.24 (s, 1H, H_c_); 6.42–8.14 (m, 18H, ArH); 9.89 (s, 1H, *N*H). ^13^C (75 MHz, CDCl_3_) 38.2, 55.4, 67.8, 71.5, 73.3, 78.3, 109.3, 120.9, 122.4, 122.6, 124.4, 126.5, 127.1, 127.2, 127.5, 127.6, 127.9, 128.1, 128.4, 133.2, 135.3, 135.9, 139.6, 140.6, 141.1, 172.4, 192.8, 199.8. EI-MS *m*/*z* 528.62 (M^+^). Anal. Calcd. for C_33_H_24_N_2_O_3_S: C, 74.98; H, 4.58; N, 5.30%. Found: C, 74.91; H, 4.65; N, 5.22%.

Hexahydro-3-phenyl-7-[(*p*-methyl)phenyl]spiro[5.2′]-2*H*-indene-1′,3′-dione-spiro[6.3″]oxindolopyrrolo[1,2-c]thiazole, 4b. Yellow solid, mp 120–122 °C; IR (KBr) 3392, 1751, 1710, 1700 cm^−1^; ^1^H (300 MHz, CDCl_3_) *δ* 2.35 (s, 3H, Me); 3.15–3.19 (m, 1H, H_d1_); 3.63–3.69 (m, 1H, H_d2_); 4.34 (d, 1H, H_a_, *J* = 9.3 Hz); 5.14–5.22 (m, 1H, H_b_); 5.46 (s, 1H, H_c_); 6.46–8.21 (m, 17H, ArH); 9.91 (s, 1H, *N*H). ^13^C (75 MHz, CDCl_3_) 26.4, 38.2, 55.4, 67.8, 71.5, 73.74 77.9, 109.2, 120.9, 122.5, 122.6, 124.4, 125.8, 127.2, 127.2, 127.5, 127.6, 128.0, 128.2, 128.4, 133.2, 135.2, 135.9, 140.5, 140.7, 141.1, 172.3, 192.9, 200.1. EI-MS *m*/*z* 542.65 (M^+^). Anal. Calcd. for C_34_H_26_N_2_O_3_S: C, 75.25; H, 4.83; N, 5.16%. Found: C, 75.19; H, 4.76; N, 5.22%.

Hexahydro-3-phenyl-7-[(*p*-methoxy)phenyl]spiro[5.2′]-2*H*-indene-1′,3′-dione-spiro[6.3″]oxindolopyrrolo[1,2-*c*]thiazole, 4c. Yellow solid, mp 128–130 °C; IR (KBr) 3389, 1748, 1712, 1691 cm^-1^; ^1^H (300 MHz, CDCl_3_) *δ* 3.12–3.17 (m, 1H, H_d1_); 3.67 (s, 3H, OMe); 3.69–3.73 (m, 1H, H_d2_); 4.40 (d, 1H, H_a_, *J* = 9.3 Hz); 5.11–5.19 (m, 1H, H_b_); 5.32 (s, 1H, H_c_); 6.42–8.27 (m, 17H, ArH); 9.96 (s, 1H, *N*H). ^13^C (75 MHz, CDCl_3_) 38.3, 55.1, 55.8, 68.5, 71.8, 73.9, 78.1, 109.5, 113.4, 122.3, 123.1, 124.9, 125.1, 127.1, 127.9, 128.1, 128.9, 129.1, 129.7, 135.6, 136.4, 139.4, 140.2, 140.8, 159.1, 172.8, 193.6, 200.3. EI-MS *m/z* 558.65 (M^+^). Anal. Calcd. for C_34_H_26_N_2_O_4_S: C, 73.10; H, 4.69; N, 5.01%. Found: C, C, 73.17; H, 4.77; N, 4.94%.

Hexahydro-3-phenyl-7-[(*p*-chloro)phenyl]spiro[5.2′]-2*H*-indene-1′,3′-dione-spiro[6.3″]oxindolopyrrolo[1,2-*c*]thiazole, 4d. Yellow solid, mp 140–142 °C; IR (KBr) 3383, 1740, 1703, 1695 cm^−1^; ^1^H (300 MHz, CDCl_3_) *δ* 3.10–3.16 (m, 1H, H_d1_); 3.66–3.73 (m, 1H, H_d2_); 4.42 (d, 1H, H_a_, *J* = 9.6 Hz); 5.12–5.20 (m, 1H, H_b_); 5.31 (s, 1H, H_c_); 6.45–8.24 (m, 17H, ArH); 8.90 (s, 1H, NH). ^13^C (75 MHz, CDCl_3_) 38.2, 55.5, 68.4, 71.6, 73.6, 77.8, 109.8, 122.4, 123.1, 123.2, 124.5, 127.1, 128.0, 128.2, 128.2, 128.9, 129.3, 129.9, 131.7, 133.8, 135.7, 136.5, 139.4, 140.1, 140.5, 140.9, 172.8, 193.4, 200.2. EI-MS *m*/*z* 563.07 (M^+^). Anal. Calcd. for C_33_H_23_ClN_2_O_3_S: C, 70.39; H, 4.12; N, 4.98%. Found: C, 70.46; H, 4.19; N, 4.94%.

Hexahydro-3-phenyl-7-[(*p*-bromo)phenyl]spiro[5.2′]-2*H*-indene-1′,3′-dione-spiro[6.3″]oxindolopyrrolo[1,2-*c*]thiazole, 4e. Yellow solid, mp 138–140 °C; IR (KBr) 3396, 1743, 1710, 1699 cm^−1^; ^1^H (300 MHz, CDCl_3_) *δ* 3.06–3.11 (m, 1H, H_d1_); 3.59–3.64 (m, 1H, H_d2_); 4.33 (d, 1H, H_a_, *J* = 9.6 Hz); 5.10–5.19 (m, 1H, H_b_); 5.41 (s, 1H, H_c_); 6.51–8.19 (m, 17H, ArH); 9.89 (s, 1H, *N*H). ^13^C (75 MHz, CDCl_3_) 38.1, 55.4, 67.7, 71.4, 72.7, 77.9, 109.4, 120.8, 122.4, 122.8, 124.0, 125.6, 127.2, 127.6, 127.7, 127.8, 128.1, 128.2, 128.5, 133.2, 135.2, 135.83, 140.5, 140.6, 141.0, 172.4, 192.8, 199.3. EI-MS *m*/*z* 607.52 (M^+^). Anal. Calcd. for C_33_H_23_BrN_2_O_3_S: C, 65.24; H, 3.82; N, 4.61%. Found: C, 65.16; H, 3.89; N, 4.56%.

Hexahydro-3-phenyl-7-[(*p*-nitro)phenyl]spiro[5.2′]-2*H*-indene-1′,3′-dione-spiro[6.3″]oxindolopyrrolo[1,2-*c*]thiazole, 4f. Yellow solid, mp 152–154 °C; IR (KBr) 3383, 1739, 1705, 1696 cm^−1^; ^1^H (300 MHz, CDCl_3_) *δ* 3.30–3.36 (m, 1H, H_d1_); 3.64–3.68 (m, 1H, H_d2_); 4.52 (d, 1H, H_a_, *J* = 9.6 Hz); 5.18–5.32 (m, 1H, H_b_); 5.52 (s, 1H, H_c_); 6.87–8.65 (m, 17H, ArH); 9.96 (s, 1H, *N*H).^13^C (75 MHz, CDCl_3_) 38.3, 55.3, 67.4, 71.6, 72.6, 78.5, 109.3, 121.3, 122.6, 123.5, 124.7, 126.9, 127.7, 127.9, 127.9, 128.0, 128.1, 128.3, 128.9, 133.7, 135.7, 136.1, 140.8, 140.7, 141.5, 172.6, 192.4, 200.6. EI-MS *m*/*z* 573.62 (M^+^). Anal. Calcd. for C_33_H_23_N_3_O_5_S: C, 69.10; H, 4.04; N, 7.33%. Found: C, 69.23; H, 4.13; N, 7.40%.

Hexahydro-3-phenyl-7-benzonitrile-spiro[5.2′]-2*H*-indene-1′,3′-dione-spiro[6.3″]oxindolopyrrolo[1,2-*c*]thiazole, 4g. Pale yellow solid, mp 112–114 °C; IR (KBr) 3380, 2247, 1726, 1710, 1690 cm^−1^; ^1^H (300 MHz, CDCl_3_) *δ* 3.33–3.38 (m, 1H, H_d1_); 3.70–3.75 (m, 1H, H_d2_); 4.45 (d, 1H, H_a_, *J* = 9.5 Hz); 5.20–5.35 (m, 1H, H_b_); 5.61 (s, 1H, H_c_); 6.99–8.77 (m, 17H, ArH); 9.93 (s, 1H, *N*H).^13^C (75 MHz, CDCl_3_) 38.3, 56.1, 67.7, 71.7, 72.7, 78.5, 110.0, 120.2, 122.3, 122.8, 123.6, 124.1, 125.9, 127.1, 128.2, 128.6, 128.9, 128.9, 129.0, 129.1, 133.7, 135.9, 136.8, 139.6, 140.1, 141.8, 173.4, 191.9, 199.9. EI-MS *m*/*z* 553.64 (M^+^). Anal. Calcd. for C_34_H_23_N_3_O_3_S: C, 73.76; H, 4.19; N, 7.59%. Found: C, 73.81; H, 4.22; N, 7.52%.

Hexahydro-3-phenyl-7-[(*p*-*N*,*N*′-dimethylamino)phenyl]spiro[5.2′]-2*H*-indene-1′,3′-dione-spiro[6.3″]oxindolopyrrolo[1,2-*c*]thiazole, 4h. Yellow solid, mp 166–168 °C; IR (KBr) 3386, 1728, 1706, 1689 cm^−1^; ^1^H (300 MHz, CDCl_3_) *δ* 3.11 (s, 6H); 3.32–3.34 (m, 1H, H_d1_); 3.51–3.59 (m, 1H, H_d2_); 4.48 (d, 1H, H_a_, *J* = 9.6 Hz); 5.21–5.32 (m, 1H, H_b_); 5.48 (s, 1H, H_c_); 6.91–8.58 (m, 17H, ArH); 9.92 (s, 1H, *N*H).^13^C (75 MHz, CDCl_3_) 36.2, 37.1, 42.7, 56.8, 66.5, 70.6, 72.2, 77.9, 110.1, 121.8, 122.1, 123.5, 124.1, 125.9, 127.3, 127.9, 128.1, 128.1, 128.2, 128.3, 130.2, 133.6, 135.8, 136.3, 139.6, 140.6, 142.7, 174.1, 191.2, 199.9. EI-MS *m/z* 571.70 (M^+^). Anal. Calcd. for C_35_H_29_N_3_O_3_S: C, 73.53; H, 5.11; N, 7.35%. Found: C, 73.26; H, 5.23; N, 7.41%.

## Conclusion

Environmentally green ACI/EG eutectic mixture mediated synthesis of novel substituted dispiropyrrolothiazole analogues through azomethineylide one-pot three component 1,3-dipolar cycloaddition reactions have been developed. This eutectic mixture mediated synthesis has the advantages of good to excellent yield, mild reaction conditions and with high regio- and stereo- selectivity. Further, the reusability of recovered eutectic mixture showed their stability and efficiency for the consecutive applications in synthesis. All the synthesized compounds were responding for commonly existing uropathogens, and **4a**, **4d**, and **4e** showed highly proficient activities compared with other compounds. In addition, MIC tests of dispiropyrrolothiazole derivatives **4a–h** were also examined by the tube dilution technique against most sensitive uropathogens viz. *K. pneumonia*, *E. coli*, and *S. aureus* and the results revealed that compounds **4d** and **4e** were effective against the uropathogens. The antioxidant activity of the synthesized compounds were assessed based on the scavenging activity of stable DPPH free radical. Interestingly, compounds **4a** (IC_50_ = 35.30 μM), **4d** (IC_50_ = 32.50 μM) and **4e** (IC_50_ = 36.80 μM) showed effective free radical inhibition better than standard inhibitor BHA (IC_50_ = 58.60 μM). With additional optimization, we trust our compounds would be promising antimicrobial drugs for to treat against uropathogens causing urinary tract infections.

## Additional file

**Additional file 1** Additional Figures and Tables.

## Abbreviations

ACI: acetylcholine iodide
EG: ethylene glycol
Bmim: 1-butyl-3-methylimidazolium
Emim: 1-ethyl-3-methylimidazolium
TLC: thin layer chromatography
FTIR: Fourier-transform infrared spectroscopy
NMR: nuclear magnetic resonance
COSY: Correlated SpectroscopY
DEPT: distortionless enhancement by polarization transfer
UTI: urinary tract infection
UPEC: uropathogenic *Escherichia coli*
MIC: mimimum inhibitory concentration
BHI: brain heart infusion
*E*. *coli*: *Escherichia coli*
*K. pneumoniae*: *Klebsiella pneumoniae*
*P*. *aeruginosa*: *Pseudomonas aeruginosa*
*S*. *aureus*: *Staphylococcus aureus*
*E*. *faecalis*: *S. epidermidis*
ATCC: American Type Culture Collection
DPPH: 1,1-diphenyl-2-picrylhydrazyl
BHA: tert-butyl-4-hydroxyanisole
DMSO: dimethyl sulfoxide
CDCI_3_: chloroform-d
EI-MS: electron ionization mass spectrometry
m/z: mass-charge ratio
