# Supplementary material for: An expeditious and environmentally benign synthesis of dispiro-3-phenylpyrrolothiazoles in ACI/EG eutectic mixture and its antioxidant and antimicrobial activities against urinary tract pathogens
Source: BMC Chem. 2019 Mar 28;13(1):42. doi: 10.1186/s13065-019-0553-3 (PMC6661829; doi:10.1186/s13065-019-0553-3)
Supplement: Supplementary file 1 — Additional file 1 Additional Figures and Tables. [file 13065_2019_553_MOESM1_ESM.docx]

**† Additional File 1**

**An expeditious and environmentally benign synthesis of dispiro-3-phenylpyrrolothiazoles in ACI/EG eutectic mixture and its antioxidant and antimicrobial activities against urinary tract pathogens**

Govindasami Periyasami^1,2,^*, Karuppiah Ponmurugan^3^, Natarajan Arumugam^1^, Raju Sureshkumar^1^, Mostafizur Rahaman^1^, Durairaju Periyan^4^, Naif Abdullah Al-Dhabi^3^, Shaykha Alzahly^1^, Ali Aldalbahi^1,^*

^1^ Department of Chemistry, College of Science, King Saud University, P.O. Box 2455, Riyadh 11451, Saudi Arabia.

^2^ Department of Organic Chemistry, University of Madras, Guindy Campus, Chennai 600 025, India.

^3^ Department of Botany and Microbiology, College of Science, King Saud University, P.O. Box. 2455, Riyadh-11451, Saudi Arabia.

^4^ Department of Chemistry, Thiruvalluar Government Arts College, Periyar University, Rasipuram, India.

Corresponding authors E-mail: [pkandhan@ksu.edu.sa](mailto:pkandhan@ksu.edu.sa), [aaldalbahi@ksu.edu.sa](mailto:aaldalbahi@ksu.edu.sa)

***Table of contents***

| S. No. | **Content** | Page |
| --- | --- | --- |
| 1 | Figure S1a: ^1^H-NMR spectrum of 4d | 3 |
| 2 | Figure S1b: ^1^H-NMR expansion spectrum of **4d** | 3 |
| 3 | Figure S1c: ^13^C-NMR spectrum of **4d** | 4 |
| 4 | Figure S2: Antimicrobial activity: Zone of inhibition of **4a-h** | 5 |
| 5 | Table S1. Antibacterial activity of dispiropyrrololhiazoles **4a-h** against uropathogens | 6 |
| 6 | Table S2. Antioxidant activity of dispiropyrrololhiazole compounds 4a-h by DPPH method | 6 |

General considerations

All melting points were uncorrected. IR spectra were recorded on a SHIMADZU IR-8300 series FT-IR spectrophotometer. ^1^H NMR and ^13^C NMR spectra were recorded on BRUKER 300 MHz instrument in CDCl_3_ and DMSO-d_6_ solvent with TMS as a standard. Mass spectra were recorded on a JEOL-DX303 HF mass spectrophotometer. Elemental analyses were carried out by Perkin-Elmer CHNS 2400B and Carlo Erba 1106 instruments. Single crystal X-ray diffraction analyses were performed by Bruker SMART APEX CCD area-detector diffractometer and Bruker SMART APEXII CCD area-detector diffractometer. Column chromatography was performed on silica gel (ACME, 100 -200 mesh). Routine monitoring of the reaction was made using thin layer chromatography developed on glass plates coated with silica gel-G (ACME) of 25 mm thickness and visualized with iodine.


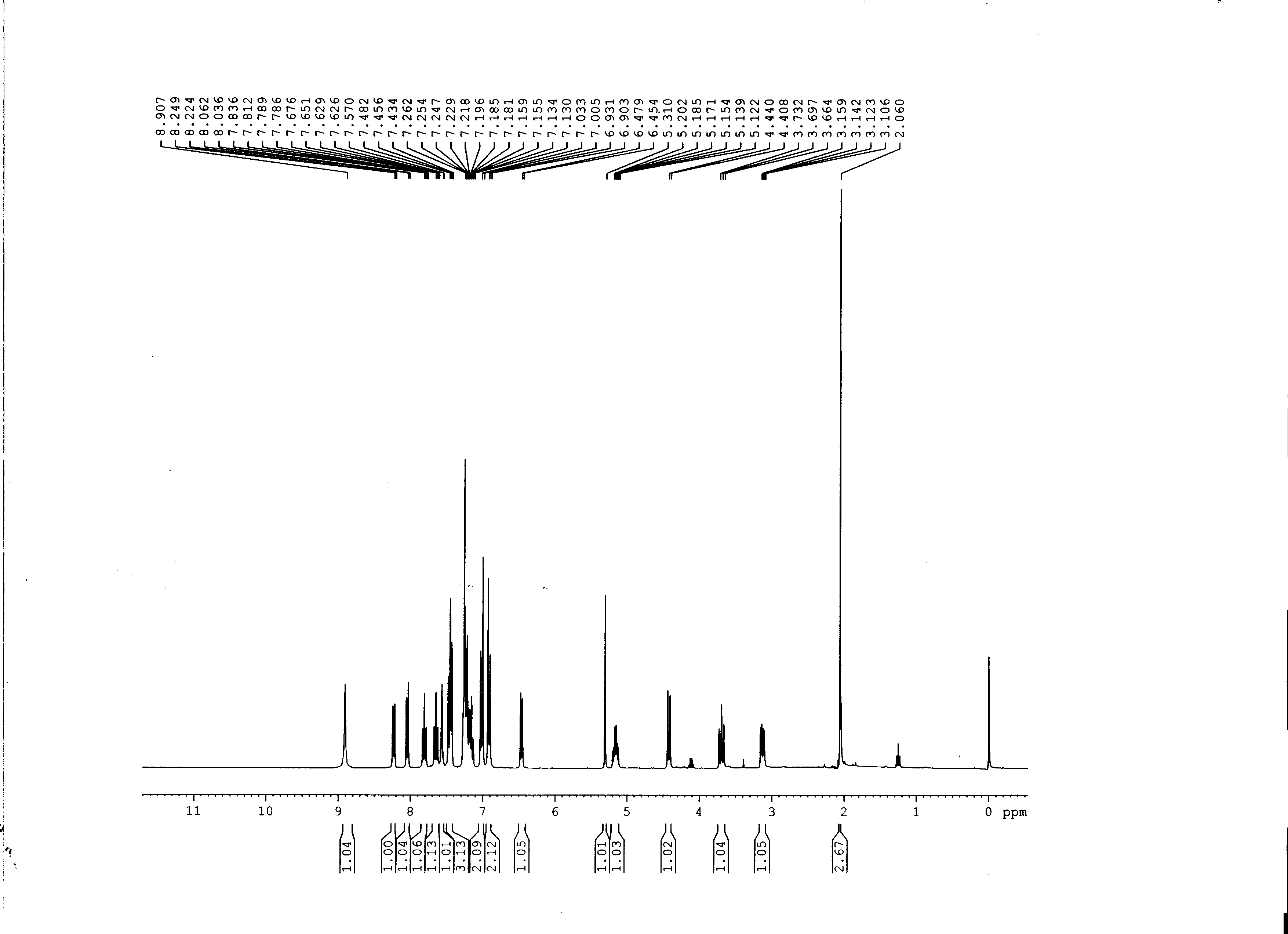


**Figure S1a**: ^1^H NMR spectrum of **4d**


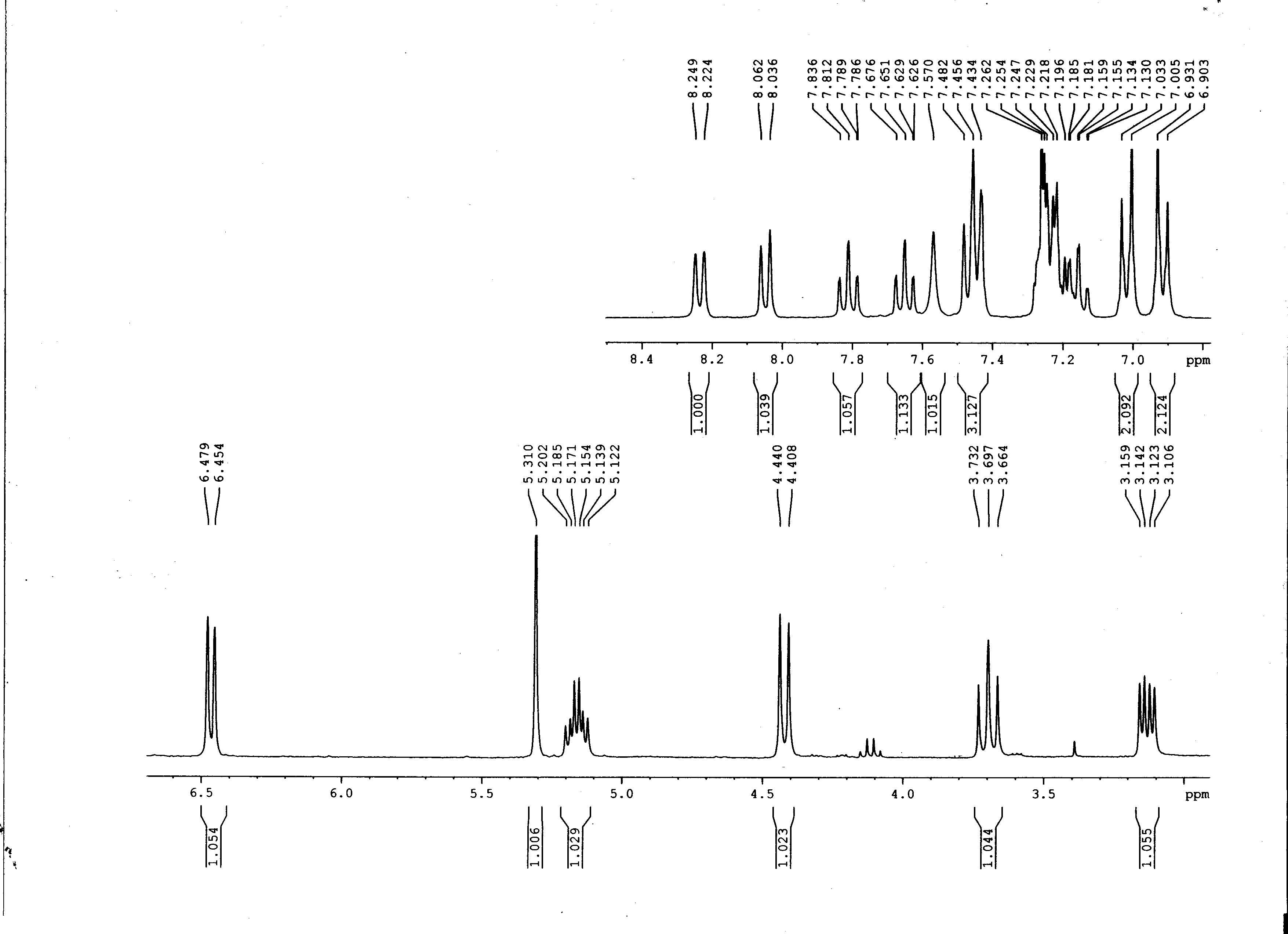


**Figure S1b**: ^1^H NMR expansion spectrum of **4d**


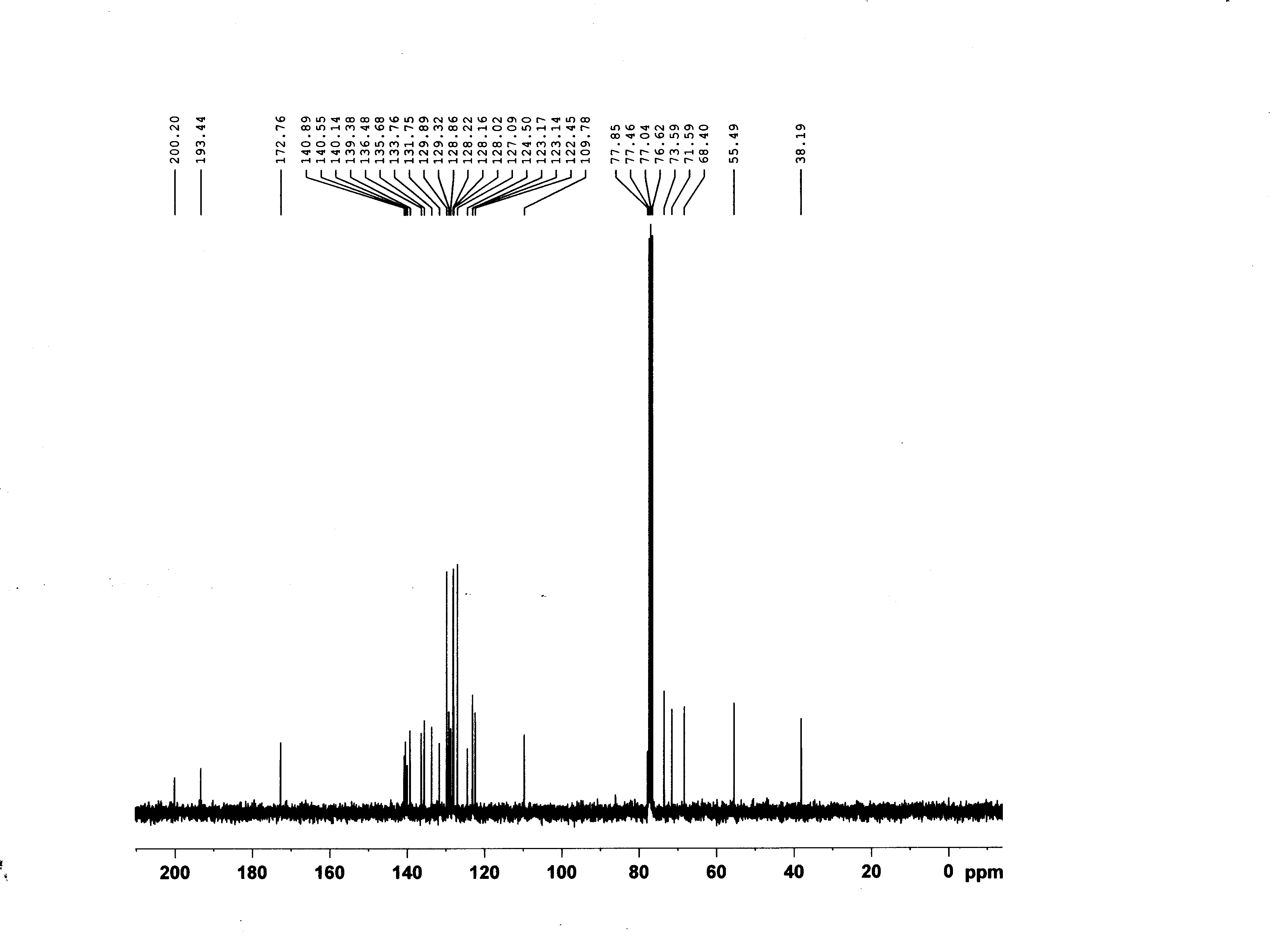


**Figure S1c**: ^13^C NMR spectrum of **4d**

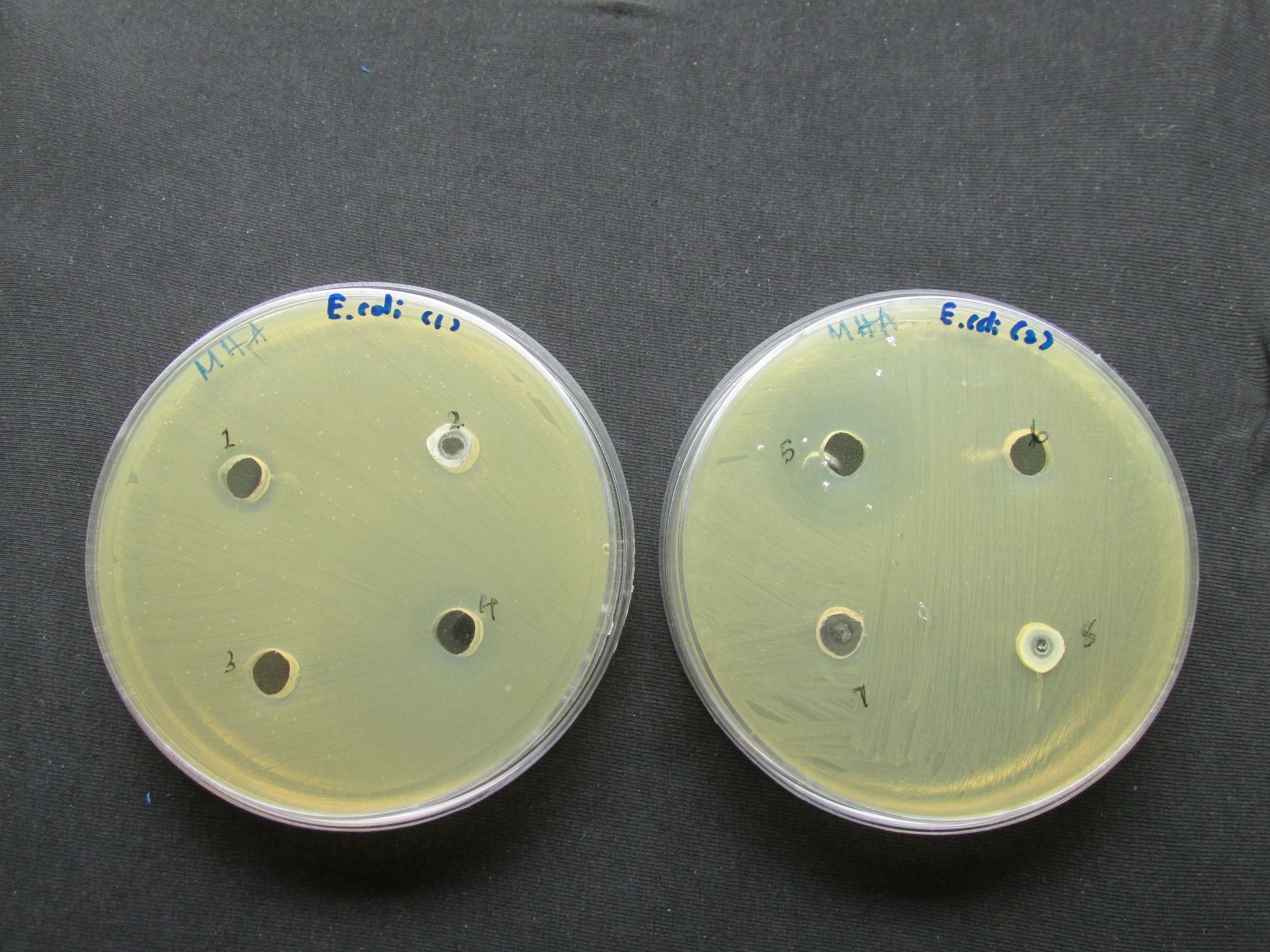


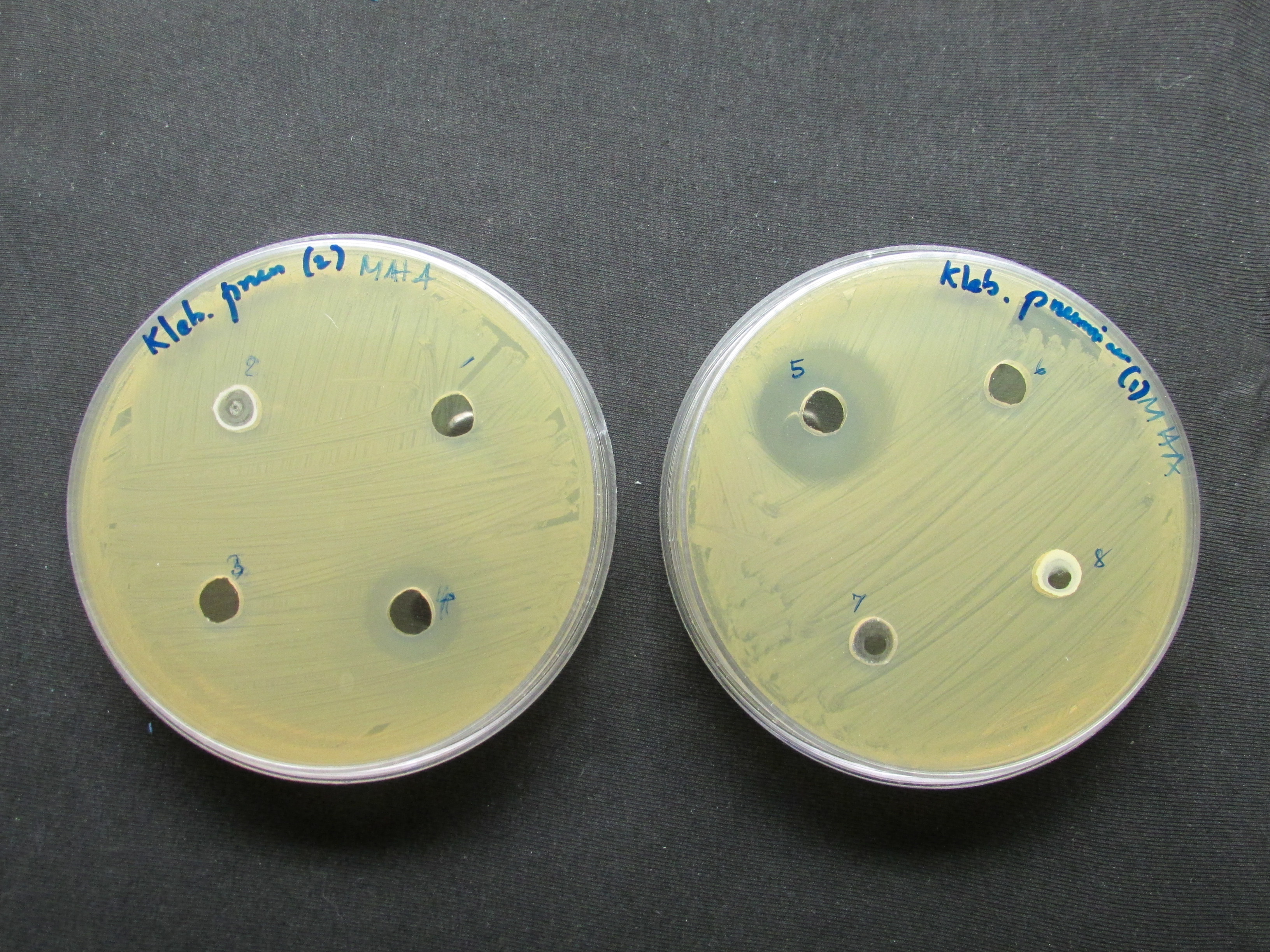

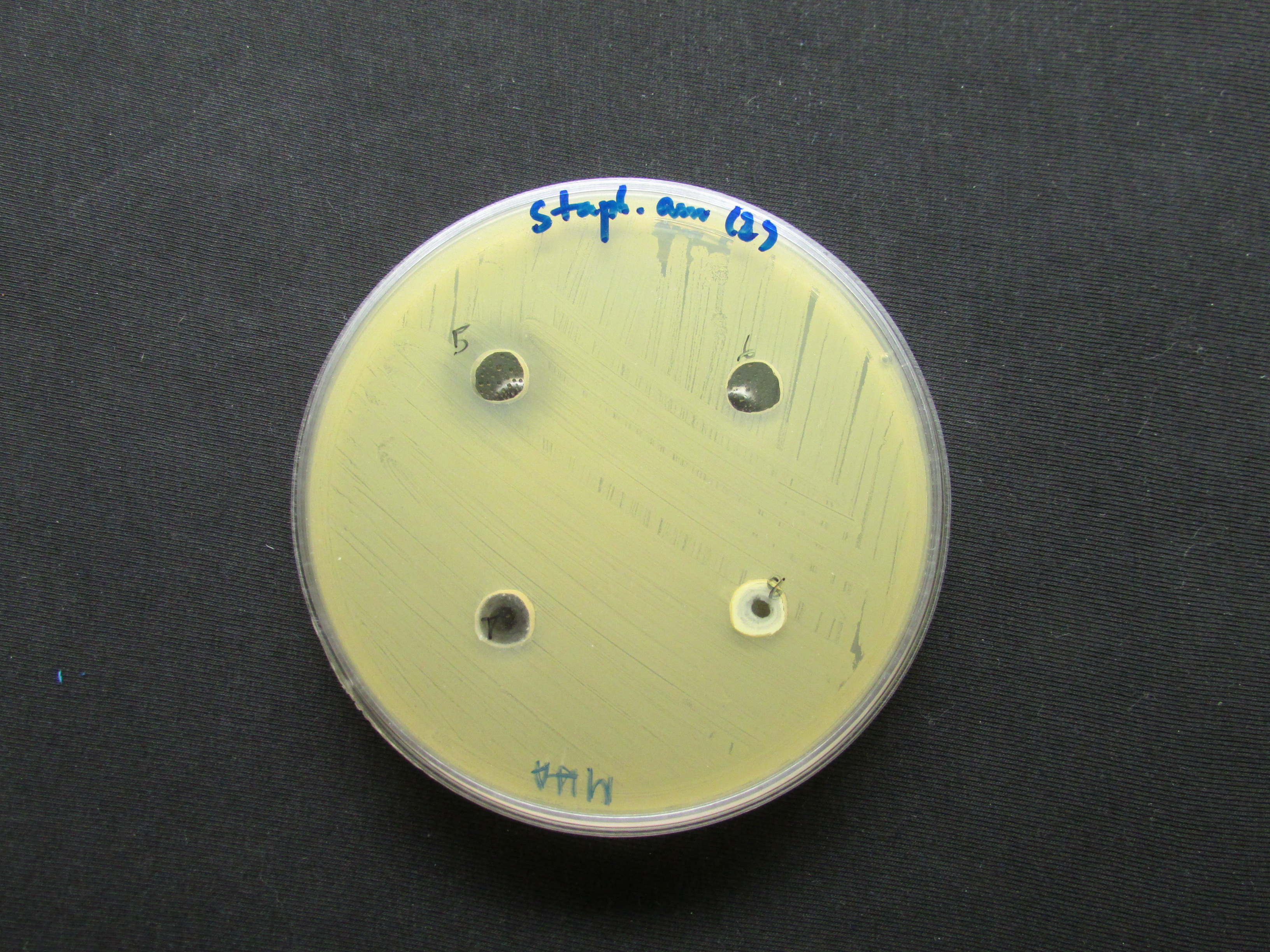


Figure S2: Zone of inhibition of 4a-h against E. *coli*, E.*coli* ATCC 25922, K. *pneumoniae*,
P. *aeruginosa*, S.*aeruginosa*, S.*aureus*, E. *faecalis*, S. *epidermidis*, S. *aureus* ATCC 29213.

**Table S1**. Antibacterial activity of dispiropyrrololhiazoles **4a-h** against uropathogens

| *Uropathogens* | 4a | 4b | 4c | 4d | 4e | 4f | 4g | 4h | Streptomycin |
| --- | --- | --- | --- | --- | --- | --- | --- | --- | --- |
| *E.Coli* | 12.50 | 07.00 | 08.00 | 14.00 | 22.00 | 07.20 | 07.00 | 08.00 | 30.00 |
| *E.Coli*  ATCC 25922 | 12.00 | 08.10 | 07.50 | 18.00 | 20.00 | 08.00 | 08.10 | 07.20 | 26.00 |
| *K. pneumoniae* | 07.11 | 07.70 | 07.20 | 15.00 | 24.00 | 07.50 | 07.70 | 07.00 | 22.00 |
| *P. aeruginosa* | 10.50 | 09.00 | 08.50 | 10.00 | 08.50 | 08.50 | 09.00 | 09.25 | 18.50 |
| *S. aureus* | 09.00 | 09.25 | 09.00 | 13.00 | 18.00 | 09.00 | 09.25 | 08.00 | 23.00 |
| *E. faecalis* | 08.10 | 07.75 | 07.00 | 7.50 | 08.00 | 07.50 | 07.75 | 07.00 | 21.00 |
| *S. epidermidis* | 07.50 | 07.10 | 07.70 | 9.30 | 07.55 | 08.20 | 07.10 | 08.70 | 17.50 |
| *S. aureus* ATCC 29213 | 10.00 | 07.50 | 07.00 | 16.00 | 14.50 | 07.70 | 07.50 | 08.00 | 24.00 |

The corresponding graphical presentation is presented in the manuscript as Figure 3

Table S2. Antioxidant activity of dispiropyrrololhiazole compounds 4a-h by DPPH method

| **Compounds** | **Scavenging Activity**  **(different concentration μM)** | | | **IC_50_ (μM)** |
| --- | --- | --- | --- | --- |
|  | 75 μM | 150 μM | 250 μM |  |
| 4a | 70.86±1.00 | 90.40±0.50 | 94.00±1.25 | 35.32 |
| 4b | 33.00±0.75 | 38.50±1.25 | 42.23±0.69 | 120.60 |
| 4c | 15.50±0.90 | 22.75±1.15 | 25.00±0.85 | 130.80 |
| 4d | 85.40±1.40 | 92.35±1.00 | 97.20±1.05 | 32.50 |
| 4e | 80.10±1.65 | 85.15±1.10 | 92.30±1.50 | 36.80 |
| 4f | 40.75±1.50 | 48.00±0.50 | 52.10±1.45 | 90.45 |
| 4g | 14.96±0.90 | 31.75±1.50 | 47.05±1.15 | 108.70 |
| 4h | 44.13±0.80 | 49.50±0.90 | 55.65±1.70 | 98.80 |
| BHA  (positive control) | 65.70±1.80 | 72.80±1.40 | 89.10±1.30 | 58.60 |

The corresponding graphical presentation is presented in the manuscript as Figure 4
